# Conserved proline residues prevent dimerization and aggregation in the β‐lactamase BlaC

**DOI:** 10.1002/pro.4972

**Published:** 2024-03-27

**Authors:** A. Chikunova, M. P. Manley, C. N. Heijjer, C. S. Drenth, A. J. Cramer‐Blok, M. Ud Din Ahmad, A. Perrakis, M. Ubbink

**Affiliations:** ^1^ Leiden Institute of Chemistry Leiden University Leiden The Netherlands; ^2^ Division of Biochemistry The Netherlands Cancer Institute Amsterdam The Netherlands; ^3^ Oncode Institute, Division of Biochemistry The Netherlands Cancer Institute Amsterdam The Netherlands; ^4^ Department of Infectious Diseases Imperial College London UK; ^5^ Zocdoc New York City New York USA; ^6^ ZoBio BV Leiden The Netherlands

**Keywords:** beta‐lactamase, conserved residues, dimerization, prolines

## Abstract

Evolution leads to conservation of amino acid residues in protein families. Conserved proline residues are usually considered to ensure the correct folding and to stabilize the three‐dimensional structure. Surprisingly, proline residues that are highly conserved in class A β‐lactamases were found to tolerate various substitutions without large losses in enzyme activity. We investigated the roles of three conserved prolines at positions 107, 226, and 258 in the β‐lactamase BlaC from *Mycobacterium tuberculosis* and found that mutations can lead to dimerization of the enzyme and an overall less stable protein that is prone to aggregate over time. For the variant Pro107Thr, the crystal structure shows dimer formation resembling domain swapping. It is concluded that the proline substitutions loosen the structure, enhancing multimerization. Even though the enzyme does not lose its properties without the conserved proline residues, the prolines ensure the long‐term structural integrity of the enzyme.

## 1 INTRODUCTION

**FIGURE 1:**
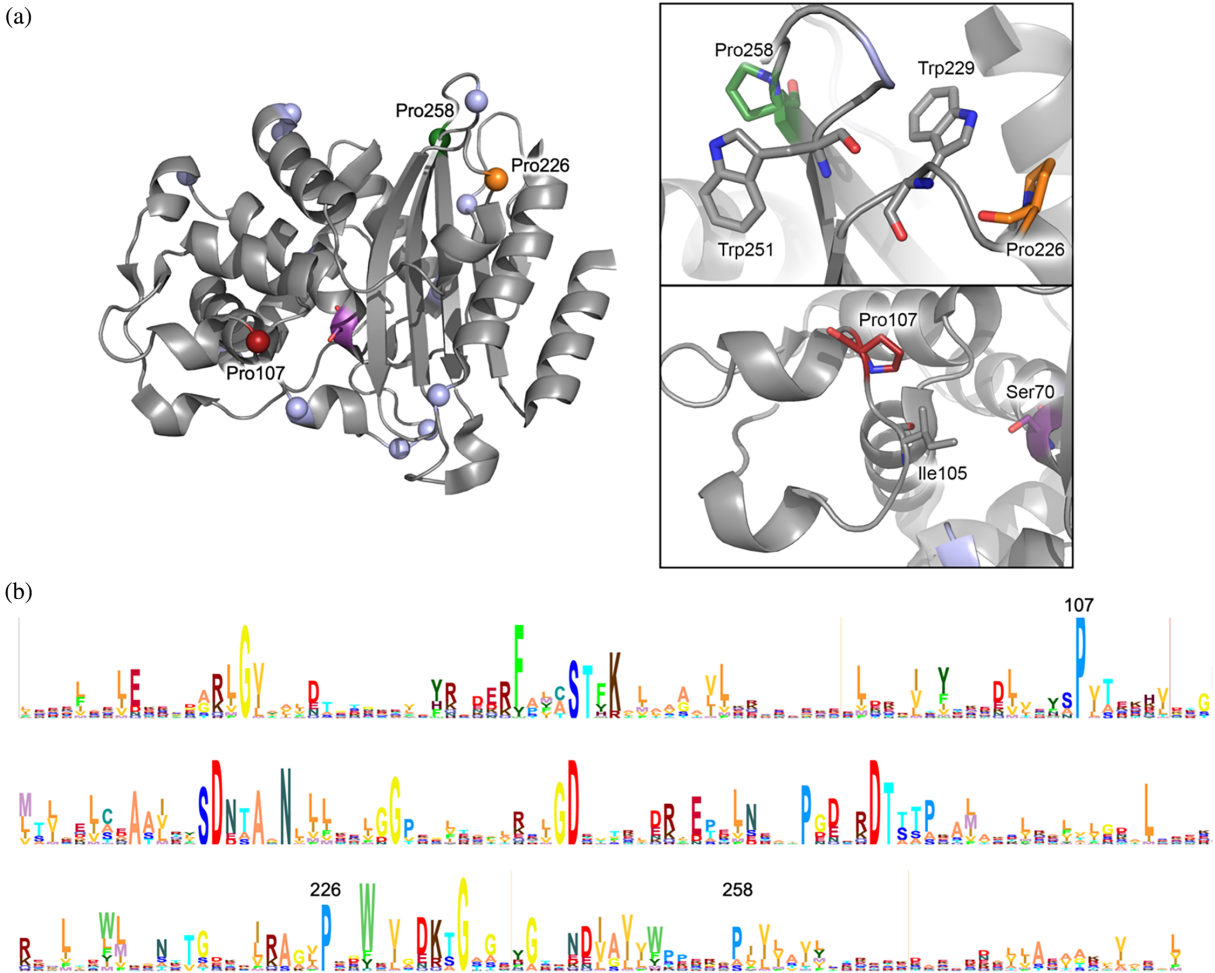
Prolines in BlaC (a) structure (2GDN (Wang et al., 2006)) and (b) consensus sequence. (a) *Left panel*: Cα atoms of proline residues are shown in spheres, with Pro107, Pro226, and Pro258 colored red, orange, and green, respectively. Ser70 is shown in purple sticks. *Right panel*: close‐ups of the regions around conserved prolines; (b) HMM logo created from multiple sequence alignment of 150 sequences of class A β‐lactamases (Schuster‐Böckler et al., 2004).

Essential amino acid residues in a protein are residues that cannot be mutated to any other residue without affecting the function (del Sol et al., 2003; Valdar, 2002). Having many essential residues makes a protein prone to function loss due to random mutations, implying a low evolutionary robustness. It is, therefore, expected that evolutionary processes tend to select proteins that function with a minimum of essential residues (Chikunova & Ubbink, 2022). In enzymes, three layers of residues can be distinguished. The residues in the active site responsible for substrate binding and catalysis form the first shell. The residues directly around the first shell tune the structure of the active site and represent the second shell. The residues in the third shell are more distant from the active site and are primarily responsible of maintaining the three‐dimensional structure by stapling the secondary structure elements in the right spatial conformation. Previously, we mutated all conserved residues in the second and third shells in the β‐lactamase from *Mycobacterium tuberculosis*, BlaC (Chikunova & Ubbink, 2022). Conservation was used as a proxy for essentiality. Many of the mutations resulted in reduced enzyme activity, either because of slight distortion of the active site (second shell) or effects on folding and stability (third shell). Interestingly, a few residues were identified for which substitutions are tolerable with marginal to no negative effect, indicating that the residues, though conserved, were not essential for BlaC (van Alen et al., 2023). Among these residues are three highly conserved prolines at positions 107, 226, and 258 (Ambler numbering) (Ambler et al., 1991). BlaC has 15 proline residues but only these three are 97%–99% conserved among class A β‐lactamases (Figure 1) (Armon et al., 2001; Ashkenazy et al., 2010; Berezin et al., 2004).

These prolines are found in the loops and are solvent‐exposed. The Cα atoms of Pro107, Pro226, and Pro258 are located 14, 25, and 23 Å away from Cα atom of catalytic residue Ser70, respectively. Pro107 is found at the edge of a small loop, which carries the so‐called gatekeeper residue Ile105, important for substrate specificity (Feiler et al., 2013). Pro107 was reported to be involved in binding of β‐lactamase inhibitory proteins (Fryszczyn et al., 2014; Rudgers & Palzkill, 1999) and antibodies (Hujer et al., 2004). Pro226 is found in a loop between β‐strand 3 and an α‐helix in an α/β‐domain, interacting with Trp229, another highly conserved residue. This interaction was shown to be important in β‐lactamase TEM‐1 for allosteric modulation of the active site, by affecting the mobility of an α‐helix (Avci et al., 2016; Meneksedag et al., 2013). Pro258 is located in a turn between β‐strands 4 and 5, stacked with Trp251, which may stabilize the turn.

The ring structure gives proline some unique properties. It is more rigid than other amino acids and can accommodate a *cis*‐conformation of the peptide bond with the N‐terminal residue. For this reason, prolines are generally found in places where a sharp turn is required and they can also introduce kinks into α‐helices (MacArthur & Thornton, 1991; Williamson, 1994). Prolines are rarely part of an active site, due to their low reactivity. They are often described to play an important role in folding (Cook et al., 1979; Kemper, 2004; Krieger et al., 2005; Levitt, 1981; Schmid & Baldwin, 1978), transmembrane domains (Hiniker et al., 2006; Joshi & Pajor, 2006; Palmieri & Pierri, 2010; van Arnam et al., 2011) or molecular recognition sites (Gupta et al., 2013; Morgan & Rubenstein, 2013), for example, at protein–protein interaction surfaces (Adzhubei et al., 2013; Bochicchio & Tamburro, 2002; Kini & Evans, 1995) or in signaling peptides (McDonald et al., 2009; Peterson & Volkman, 2009).

Given the high conservation and chemical nature of the residues at the positions 107, 226, and 258, the limited negative effect of the substitutions is rather unexpected. In this study, we investigated the effects of substitutions on the structure, activity, and stability of BlaC. It was found that substitutions in all three prolines lead to an overall less stable enzyme, more prone to oligomerization and aggregation. The crystal structure of BlaC P107T suggested a mechanism of dimer formation for this variant.

## 2 RESULTS

### 2.1 Most substitutions in conserved prolines have a marginal effect on activity and thermostability

**FIGURE 2:**
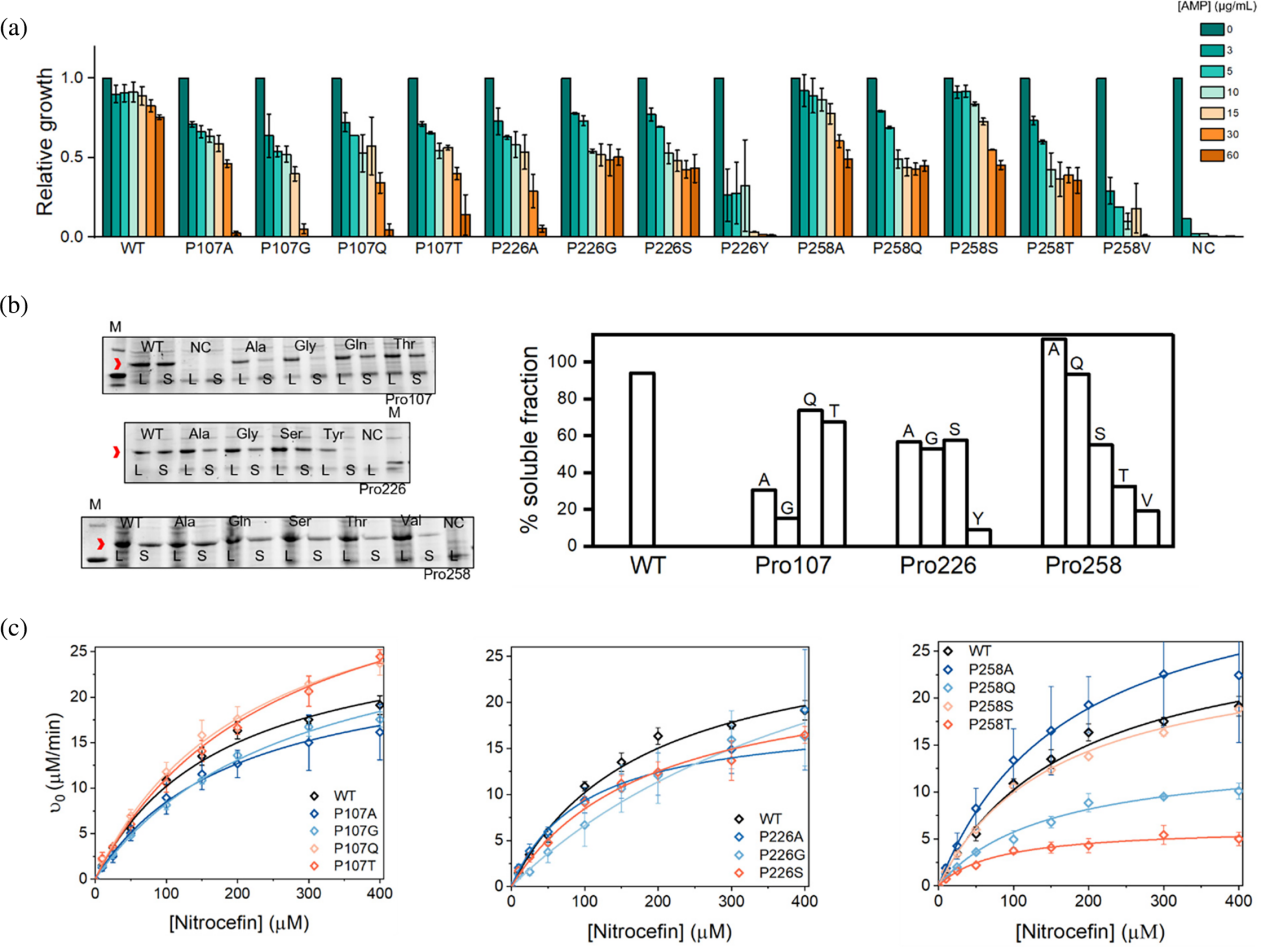
Activity of proline variants of BlaC. (a) *E. coli* cell growth in presence of different concentrations of ampicillin relative to growth without antibiotic, determined as OD of liquid cultures after 18 h incubation at 37°C. Error bars represent standard deviation of two biological replicates; (b) SDS‐PA‐gels showing the presence of BlaC variants (indicated by the red arrows) in whole‐cell lysate (L) and soluble fraction (S), NC = negative control (*left panel*); Percentage of soluble protein derived from the comparison of the signal intensities from SDS‐PA‐gels, the error of determination is 10% (*right panel*); (c) Michaelis–Menten curves of reactions of substrate nitrocefin degradation. Error bars represent the standard deviation of three replicates, the lines represent fits to the Michaelis–Menten equation, *v*_0_ = *V*_max_[S]/(*K*_M_+[S]), where *ν*_0_ and *V*_max_ are the initial and maximal conversion velocity, S is the initial substrate concentration and *K*_M_ is the apparent Michaelis constant.

Single point mutations were introduced to prolines at positions 107, 226, and 258. Substituting amino acids without (Gly) or polar (Ser, Thr, Gln), apolar (Ala, Val), and aromatic (Tyr) side chains were selected to allow for different types of interactions. To characterize the degree of resistance conferred by the BlaC variants, *Escherichia coli* cells producing the BlaC variants with a TAT signal peptide for transport to the periplasmic space (Chikunova & Ubbink, 2022) were incubated both on plates and in liquid cultures containing ampicillin or carbenicillin. All variants clearly demonstrate reduced ability to degrade either antibiotic, in line with the essential nature of the three prolines suggested by their conservation. However, most variants still show sufficient β‐lactamase activity to allow for bacterial growth even at high concentrations of antibiotics, with the exception of BlaC P226Y and BlaC P258V. *E. coli* cells producing BlaC P258A or P258S demonstrated the best growth with either antibiotic, although still somewhat less than wild type (Figures 2a, S1). In contrast, our previous study on all conserved, non‐active site residues showed that for the vast majority of the substitutions in conserved residues, the survivability of the cells dropped to the negative control level (represented by the inactive variant BlaC S70A). Thus, such a limited effect of almost all substitutions in conserved prolines on bacterial growth is remarkable.

The solubility of the BlaC variants in cells was assessed with a T7 promotor cytoplasmic overexpression system in *E. coli* (van Alen et al., 2023). For BlaC P107V and P226Y, the production of soluble protein is very low, while for the other variants, it was at most slightly lower than for wild type (Figure 2b). The soluble variants were produced and purified and those that yielded folded protein (as evaluated with CD spectroscopy, Figure S2) were further investigated in vitro. The thermostability of the BlaC variants was evaluated with two methods (Figure S2). Change in tryptophan fluorescence upon unfolding reports on the solvent accessibility of the three Trp residues in BlaC–Trp210, Trp229, and Trp251, of which the latter two interact with Pro226 and Pro258 respectively. The melting temperatures (*T*_m_) of BlaC P107 variants were 4–6 degrees lower than for wild‐type BlaC (Tables 1, S1). Interestingly, Gly and Ala substitutions cause a larger destabilization than Thr and Gln, though the latter are larger residues. Gly and Ala may introduce more conformational freedom around the mutation site, reducing thermostability. For Pro226 variants, *T*_m_ values were about 2 degrees lower than for wild type, and for Pro258 variants, *T*_m_ changes were small, indicating that both these residues are unlikely to be essential for the thermal stability. The second method uses a change in fluorescence of a fluorescent compound upon binding to the (partially) unfolded protein. Although the absolute melting temperatures obtained with the two methods differ, the observed changes in *T*_m_ were similar, with some more differences between the methods for Pro226 and Pro258, possibly due to the altered interaction with either one of the tryptophan residues.

**TABLE 1:** Kinetic parameters for the hydrolysis of nitrocefin and difference in the melting between wild type and mutant BlaC.

	Nitrocefin activity			Thermostability (Trp fluorescence)	Thermostability (hydrophobic dye)
	*k*_cat_ (s^−1^)^a^	*K*_M_ (μM)^a^	*k*_cat_/*K*_M_ (10^5^ M^−1^ s^−1^)^a^	Δ*T*_m_ (°C)^b^	Δ*T*_m_ (°C)^b^
WT	95 ± 5	178 ± 15	5.3 ± 0.5	(62.0 ± 0.1)	(51.92 ± 0.03)
P107A	77 ± 3	165 ± 13	4.7 ± 0.4	−6.1 ± 0.1	−5.0 ± 0.1
P107G	104 ± 7	275 ± 22	3.7 ± 0.4	−6.3 ± 0.2	−6.1 ± 0.1
P107Q	119 ± 3	238 ± 11	5.0 ± 0.3	−4.6 ± 0.1	−4.5 ± 0.3
P107T	140 ± 7	298 ± 28	4.7 ± 0.5	−4.1 ± 0.1	−3.9 ± 0.1
P226A	67 ± 16	113 ± 30	5.9 ± 0.2	−2.9 ± 0.1	−4.0 ± 0.2
P226G	97 ± 19	282 ± 25	3.4 ± 0.7	−2.2 ± 0.1	−1.6 ± 0.1
P226S	80 ± 7	186 ± 29	4.3 ± 0.8	−2.1 ± 0.2	−2.1 ± 0.3
P258A	118 ± 7	175 ± 12	6.7 ± 0.6	1.6 ± 0.2	0.8 ± 0.1
P258Q	48 ± 4	160 ± 28	3.0 ± 0.6	−1.4 ± 0.2	−2.5 ± 0.1
P258S	86 ± 3	158 ± 11	5.4 ± 0.4	0.7 ± 0.1	−0.1 ± 0.0
P258T	22 ± 5	84 ± 41	2.5 ± 1.3	−1.3 ± 0.1	−3.4 ± 0.1

*Note*: Values in brackets indicate the melting temperatures of wild type BlaC determined with both methods.

^a^ Errors represent standard deviation of a triplicate measurement.

^b^ Errors represent propagated errors.

Interestingly, substitutions only had marginal effect on the nitrocefin activity of BlaC variants. Catalytic efficiencies (*k*_cat_/*K*_M_) of all BlaC variants were similar, with the lowest one detected for BlaC P258T, being only two‐fold lower than that of BlaC wild type (Figure 2c, Table 1). Almost all Pro107 mutants displayed somewhat increased *K*_M_ values. Generally, Pro226 and Pro258 variants were less affected by the mutations in terms of both thermal stability and soluble protein production than Pro107 variants, correlating with the activity in the cellular assays. Given that catalytic activity of all variants was not affected greatly, we conclude that the slight negative effect on the activity in cells is likely to be mostly caused by the reduced amount of folded enzyme. This observation is in line with the finding that changes in residues far from the active site can reduce the level of the soluble enzyme but the folded form functions as the wild‐type enzyme (Chikunova & Ubbink, 2022).

### 2.2 Substitutions lead to an overall less stable enzyme prone to aggregate

**FIGURE 3:**
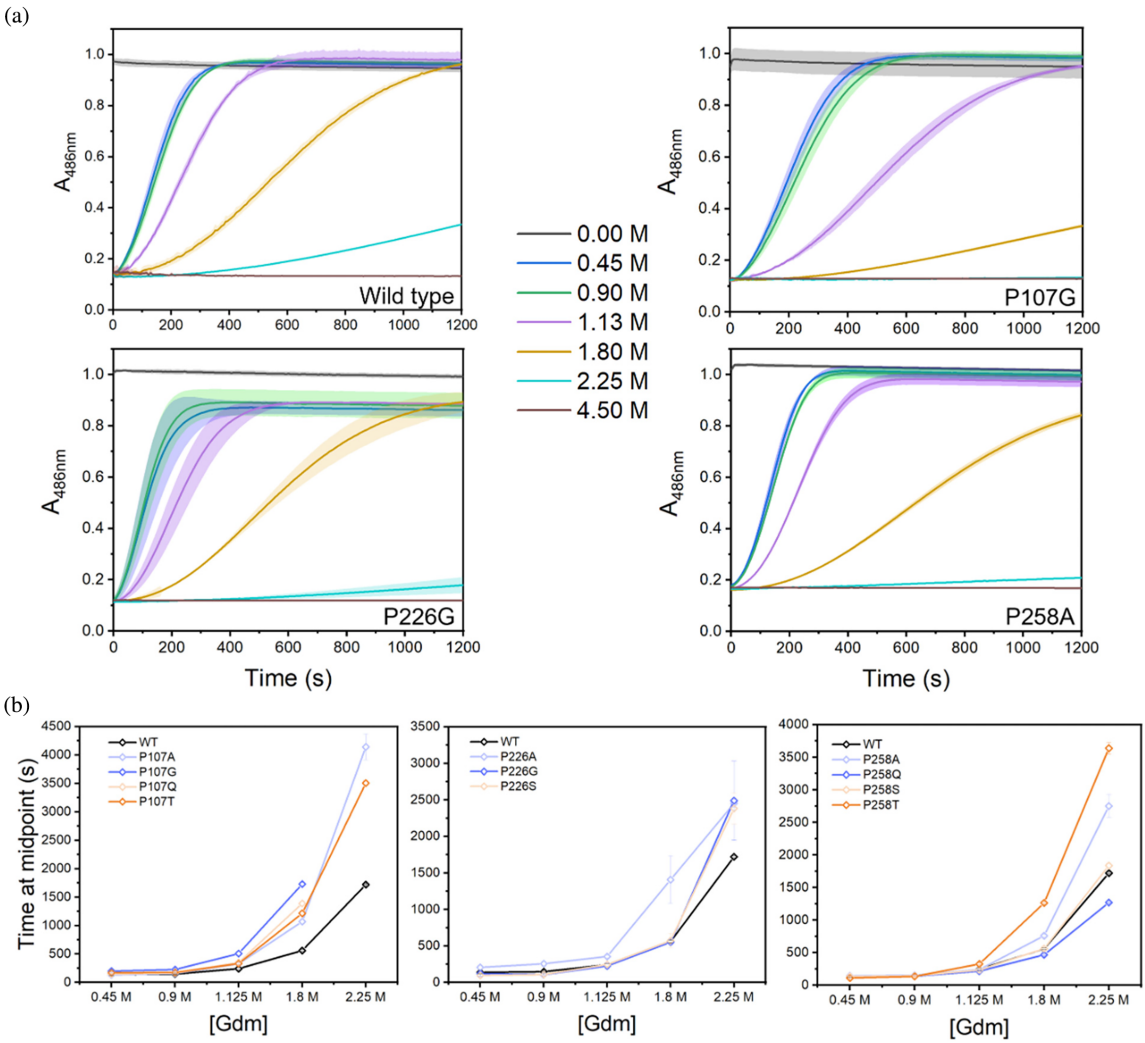
Refolding experiments. (a) Nitrocefin conversion (absorbance at 486 nm) as a function of time is shown for BlaC wild type and variants fully denatured in 4.5 M guanidinium chloride (Gdm) and diluted at *t* = 0 s to the indicated concentrations. Curves represent the average of a triplicate measurement with standard deviations shown as highlights; (b) The midpoint times derived from the results from the panel (a) by fitting product formation curves to a sigmoidal. Error bars represent the standard deviation of a triplicate measurement. For P107G and P107Q the times at 2.25 M Gdm could not be derived due to a very low activity.

The refolding ability of all variants was assessed after denaturation with chaotropic agent. The enzymes were denatured in 4.5 M guanidinium chloride (Gdm) and diluted to lower the Gdm concentration. Refolding was monitored by determining activity as measured by nitrocefin hydrolysis. All BlaC variants except BlaC P258Q demonstrate a lower refolding ability than wild‐type BlaC, which indicates that either for mutants a larger fraction of enzyme is not able to fold correctly, or folding is slowed down (Figure 3a, b). These results suggest that the three prolines play a role in rapid, proper folding, although it is noted that folding de novo on the ribosome may not be directly comparable to the refolding after chemical unfolding.

**FIGURE 4:**
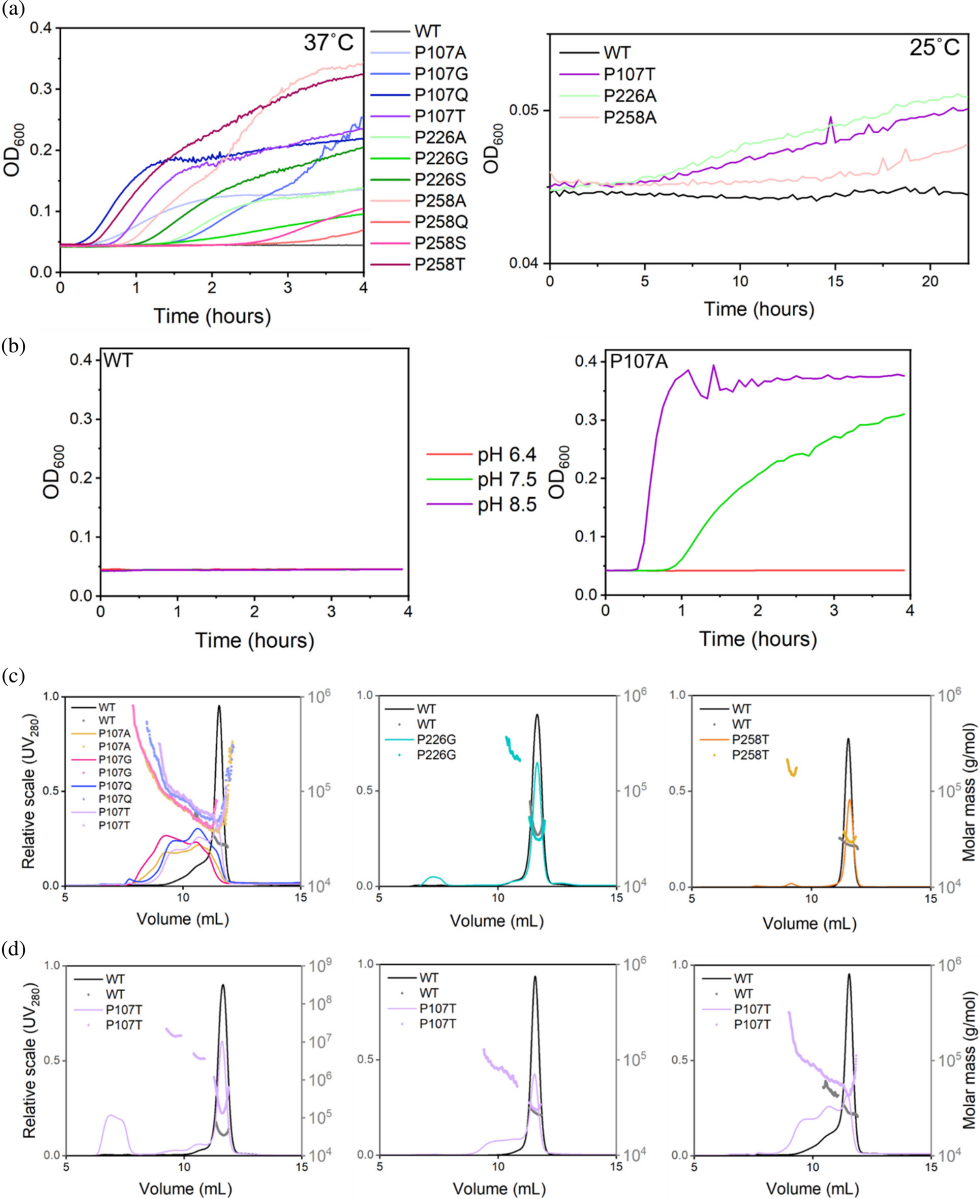
Aggregation assays. (a) Formation of aggregates as determined by a scattering signal at 600 nm at 37°C in PBS buffer pH 7.5 (left panel) or at 25°C in sodium phosphate buffer pH 6.4 (right panel); (b) The pH dependence of the scattering signal at 37°C in sodium phosphate buffer pH 6.4 (100 mM) as a measure of aggregates formation. *Left*, wild type BlaC, *right*, BlaC P107A; (c) and (d) SEC‐MALS profiles of BlaC wild type and variants in PBS buffer pH 7.4. Calculated molar masses are shown as scatter. Void volume of the column was 6.5 mL. (c) All BlaC mutants display the formation of aggregates. (d) The examples of variations of sample composition between preparations. Samples presented in each graph contained the same amount of protein.

The stability over time for BlaC wild type and mutants at 37 and 25°C was also determined. The formation of aggregates was followed by measuring absorption of light due to scattering at 600 nm. Two buffers were used, 100 mM sodium phosphate buffer (pH 6.4), which is generally used as the optimal buffer for BlaC, and phosphate‐buffered saline or PBS buffer (pH 7.4), which better resembles the expected physiological conditions. The wild‐type BlaC remained stable in both buffers and at both temperatures during the duration of the experiment (4 h at 37°C and 22 h at 25°C). All mutants also remained stable in sodium phosphate buffer pH 6.4 for 4 h at 37°C, and at 25°C only a weak OD_600_ signal was detected after 5–14 h (Figure 4a). In PBS buffer pH 7.4 at 37°C, the mutant enzymes started to show formation of aggregates within 15–180 min (Figure 4a), suggesting that the increased pH has a negative effect on stability. In phosphate buffer in the pH range 6.4–8.5, no effect on the stability of the wild‐type enzyme was observed, whereas a pH higher than 7.5 clearly influences the state of the BlaC variants (Figure 4b). At 25°C in PBS buffer, all mutants except BlaC P226G and P226S showed some formation of aggregates, but to a lesser extent than at 37°C and with wider range in lag time (Figure S3a).

Size exclusion chromatography (SEC) coupled to multiangle light scattering (MALS) detection demonstrated the presence of the dimers and possibly trimers or higher‐order species for the variants that showed aggregation in the scattering tests (Figure 4c). For some samples of the Pro107 variants, no peak with the molecular weight of the monomer was detected. Instead, a peak with molecular weight lower than dimer but higher than monomer was observed, suggesting an equilibrium between monomeric and dimeric states. Interestingly, for the Pro226 and Pro258 mutants, the MALS determined molecular weight was also higher than that of a monomer (Table S2), even for the samples showing a single peak with the elution time of the monomer, pointing toward the possible exchange between a dominant monomeric form and a dimer form with a low population. For most variants, species of very high molecular weight were detected, indicating aggregation. All described populations were observed when the column was equilibrated and eluted with PBS buffer pH 7.4, regardless of the buffer in which the samples were stored prior to the experiment. Thus, the formation of the higher order species is a relatively fast process. Equilibrating the column with phosphate buffer pH 6.4 resulted in single monomeric peak for all BlaC variants (Figure S3b). Moreover, the extent of aggregation in PBS buffer varied between preparations, even without any changes in the protocol procedure (Figure 4d).

### 2.3 BlaC P226G and P258A exhibit limited structural changes

**FIGURE 5:**
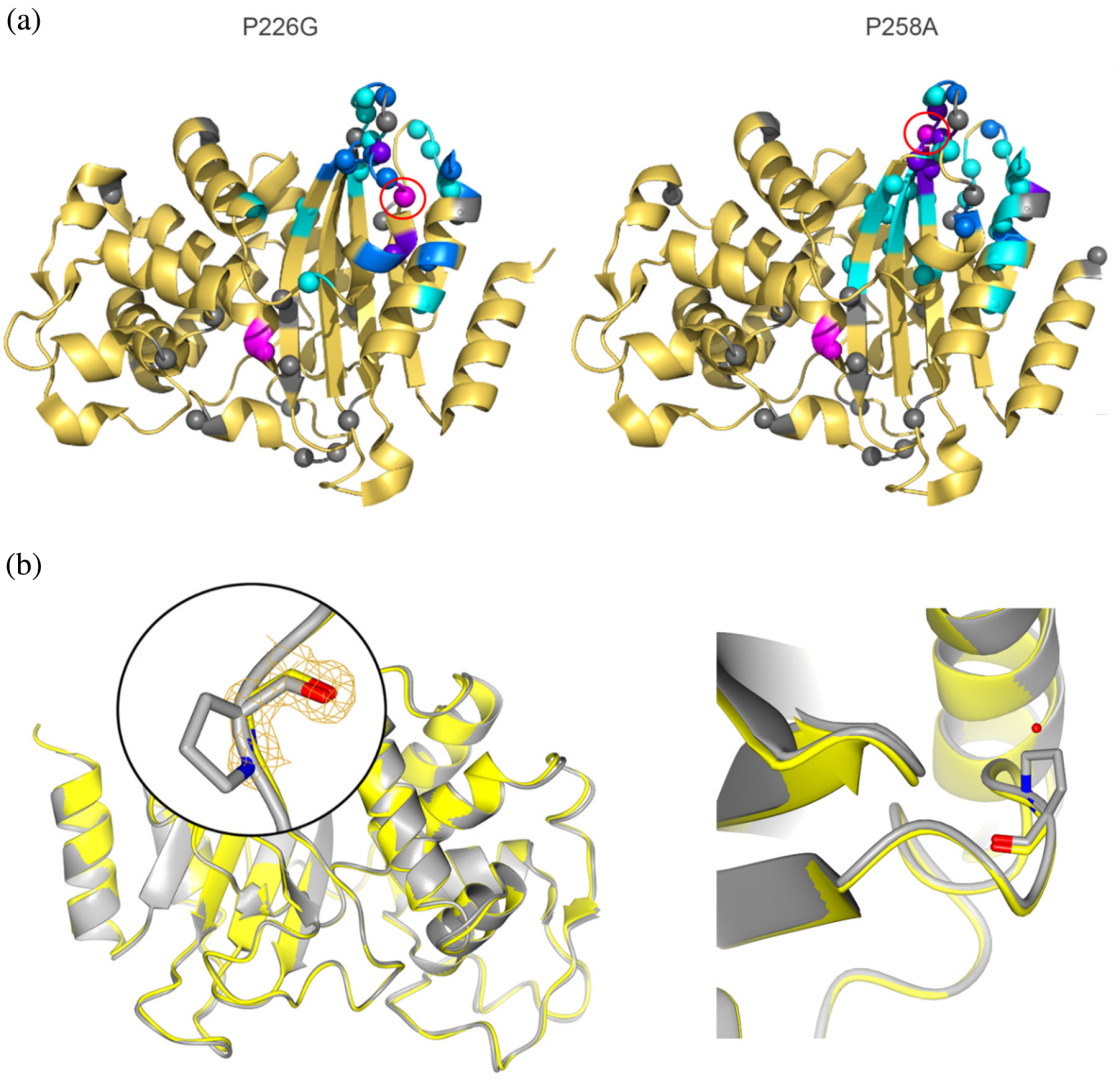
Structural analysis. (a) Average CSPs between the amide resonances of BlaC P226G and wild type BlaC mapped on the structure of BlaC P226G (7A6Z, left panel) and between BlaC P258A and wild type BlaC mapped on the wild type structure (2GDN (Wang et al., 2006), right panel). Residues are colored cyan for CSP >0.025 ppm; blue for CSP >0.05 ppm and purple for CSP >0.1 ppm and gray for no data (comprizing proline residues and some active site residues). Ser70 and mutated residues are shown in magenta. Red circles indicate the mutation sites. Relevant backbone nitrogen atoms are shown as spheres. (b) Overlay of wild type BlaC (gray) and BlaC P226G (yellow) crystal structures. The 2mFo‐DFc electron density map is shown in yellow chicken wire with contour level of 1 σ and extent radius of 5 Å. Right panel shows the close up of the mutation site with an extra ordered water molecule in BlaC P226G.

To probe the effects of the mutations on the structure of the protein, TROSY‐HSQC spectra were recorded with ^15^N labeled mutants. All mutants show changes in chemical environment that are mostly located around the mutation site (Figure 5a), indicating that structural changes introduced by substitution are limited to specific sites. The crystal structure of BlaC P226G solved to 1.3 Å displays virtually no differences to the structure of wild‐type BlaC, with average Cα RMSD of 0.34 Å (Figure 5b). Surprisingly, even the loop containing residue 226 displayed no changes. The absence of the pyrrolidine ring allowed for an additional ordered water to be present in a mutant structure.

### 2.4 BlaC P107T forms a stable dimer

**FIGURE 6:**
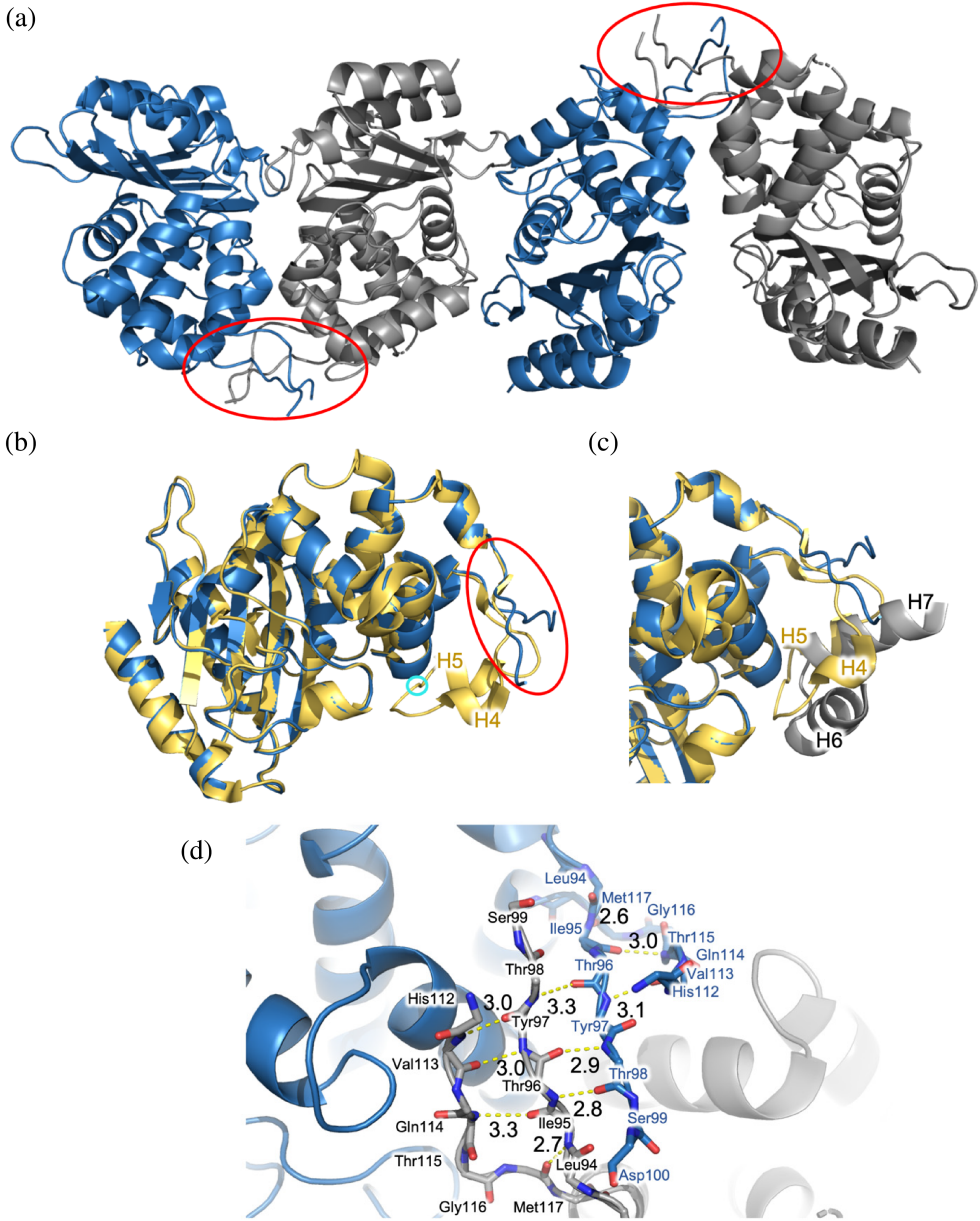
Crystal structure of BlaC P107T (8R88). (a) Four molecules are found in the asymmetric unit, forming two sets of dimers (the interacting parts are shown within red circle); (b), (c) Overlay of BlaC P107T in blue and wild type BlaC (2GDN (Wang et al., 2006)) in yellow. The red circle shows the part interacting with the second molecule; position of Pro107 in wild type BlaC is shown with cyan circle. (c) Part of the one molecule of BlaC P107T (gray) occupies the space in the other molecule which is taken by helices 4 and 5 in the structure of wild type BlaC. In the mutant these two helices are restructured and partly disordered. (d) The interacting regions (indicated with the red circle on panel a) of two BlaC P107T molecules are shown. Distances between backbone oxygen and nitrogen atoms are indicated and potential H‐bonds are shown as yellow dashed lines.

X‐ray crystallography suggests a potential mechanism of dimer formation. The crystal structure of BlaC P107T was solved at 1.9 Å with an asymmetric unit comprising four molecules forming two sets of dimers (Figure 6a). The interface area of both sets of dimers was calculated to be 1142 Å^2^, while the interface area of a monomeric wild type structure (2GDN (Wang et al., 2006)) is 485 Å^2^. Overall, the mutant structure resembles the structure of the wild type with an average Cα RMSD of 0.28 Å, excluding the disordered region, (Figure 6b). However, large differences are present near mutation site. The region around the mutation (residues 94–117), including α‐helices 4 and 5, are restructured. Residues involved in the structural changes comprise about 7 percent of the whole sequence. Eleven residues (101–111), could not be solved due to disorder. The space normally filled by α‐helices 4 and 5 in the structure of the wild‐type enzyme is occupied by α‐helices 6 and 7 of the other molecule of the dimer pair (Figure 6c). Residues 94–100 and 112–117 interact with each other within one enzyme molecule and with the same stretches in another enzyme molecule, forming an anti‐parallel β‐sheet (Figure 6d) and stabilizing the dimer. Interestingly, the crystals were formed in 50:50 ratio of phosphate buffer with pH 6.4 and malonic acid buffer with the same pH, proving that the formation of dimers is not only occurring at pH >7.5, as was observed in solution. The crystal was formed in significantly longer time (>2 months) than is usual for most BlaC variants (3–7 days), suggesting that crystallization selected a specific conformer.

**FIGURE 7:**
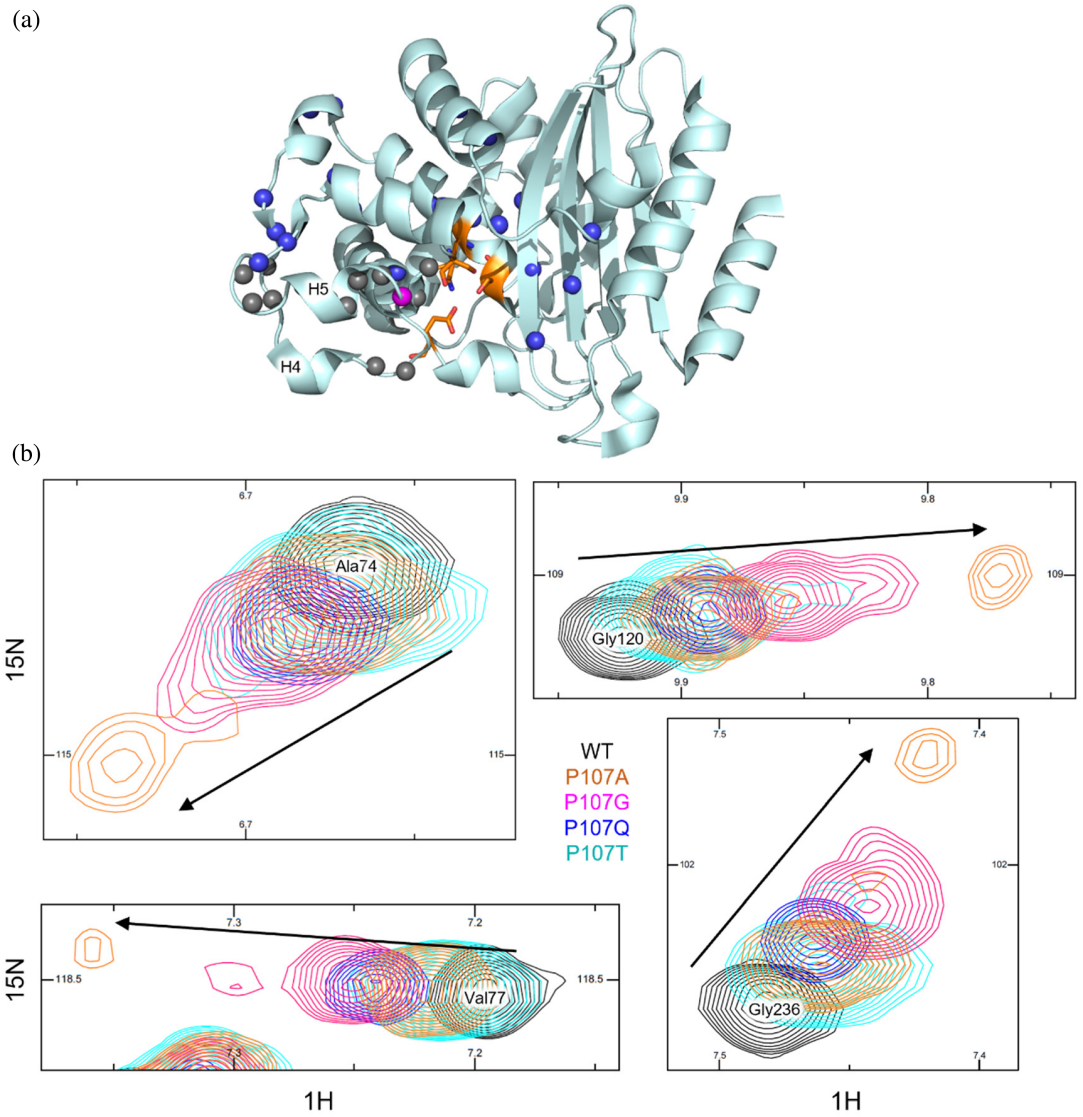
NMR spectra of BlaC P107 variants. (a) Crystal structure of wild type BlaC (2GDN (Wang et al., 2006)) indicating the amide nitrogen atoms with resonances showing a linear shift for different mutants as blue spheres. The nitrogen atom of P107 is shown as a magenta sphere. Amides with peaks broadened beyond detection in Pro107 mutants are shown as gray spheres. Several active site residues are shown in orange sticks. (b) Examples of peak doubling for BlaC P107A and P107G as well as the linear shift patterns of peaks for different mutants.

Similar to Pro226 and Pro258 mutants, variants of Pro107 display localized effect on the structure as judged by NMR spectroscopy. Close to the mutation site amide resonances were broadened beyond detection. Throughout the substrate binding pocket, amide peaks of the Pro107 mutants were detected in a linear trend shifting from the resonance position of the wild‐type BlaC toward the peak of BlaC P107G (Figure 7). The shifts of the peaks were accompanied by the broadening of the signal or double peaks for BlaC P107A and P107G (Figure 7). Based on the observations in the crystal structure, we assume the exchange detected by NMR to be the combination of multiple processes. The conformational change of the region comprising α‐helices 4 and 5 from closed to open, could be a process in the fast exchange regime. The interactions that lead to formation of the β‐sheet and dimerization could represent a much slower process.

## 3 DISCUSSION

Substitution of highly conserved residues in BlaC in most cases leads to drastic effects on the enzyme (Chikunova & Ubbink, 2022). Mutation of three conserved prolines, Pro107, Pro226, and Pro258, however, had a modest effect on BlaC activity in vitro and in bacteria. In the current study, the aim was to establish the possible role(s) of these prolines and the reason for their conservation among class A β‐lactamases. Most substitutions in the Pro107 and Pro226 resulted in slightly decreased thermal stability. All tested Pro258 variants, as well as some for Pro226 and Pro107, displayed lower yield of soluble protein in the cytoplasmic expression system in *E. coli*. BlaC P258V was mostly insoluble. The stability over time of purified enzyme at ambient temperature or 37°C as well as the refolding ability were decreased for variants of all three prolines, compared to the wild type enzyme, and dimers or higher order aggregates were detected with SEC. These observations suggests that substitution of the prolines tends to shift the balance from folding to aggregation.

**FIGURE 8:**
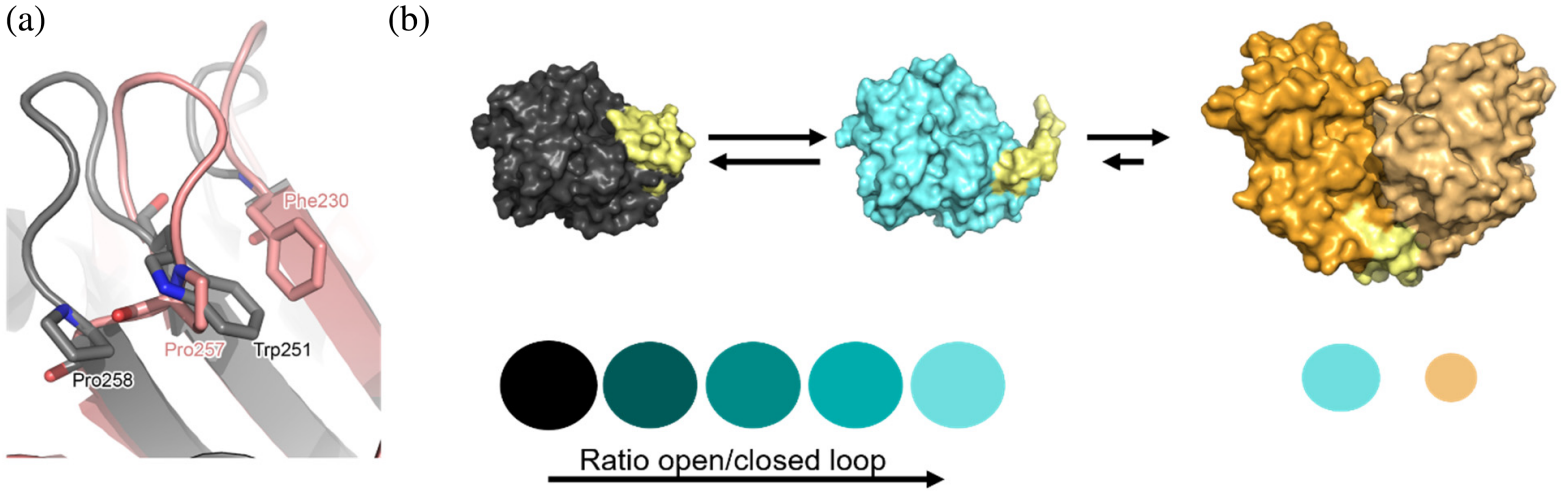
Structural effects. (a) Overlay of BlaC structure in gray (2GDN (Wang et al., 2006)) and TEM structure in pink (1ZG4 (Stec et al., 2005)). (b) Proposed model of the exchange between closed and open loop conformation with subsequent formation of the dimers for Pro107 mutants, with the effects on the amide peaks shown at the bottom in matching colors.

The fraction of the protein that is folded has a phenotype very similar to that of wild‐type BlaC. For BlaC Pro226 variants, the structures closely resemble that of wild‐type BlaC. Changes in NMR resonances of amides were localized around the mutation site and virtually no changes were found in the crystal structure. It is plausible that this highly conserved proline located in the loop limits protein dynamics. For example, substitution of proline to glycine has been shown to increase the affinity for a ligand in the human Lck SH3 domain. The variant exhibited increased loop flexibility and enhanced sampling of binding‐competent conformations (Bauer & Sticht, 2007). In TEM‐1, the stacking interaction between conserved residues Pro226‐Trp229‐Pro252 was proposed to be important for modulating the H10 helix mobility (Meneksedag et al., 2013). Similarly to Pro226 variants, for substitution P258S the effects were minimal. Interestingly, one of the best‐studied class A β‐lactamases, TEM‐1, has a serine in this position naturally (Stec et al., 2005). The relevant loops are oriented differently in TEM‐1and BlaC (Figure 8a). TEM‐1 carries a proline at the position 257, which interacts with Phe230, located within the B3 β‐strand of the protein, similar to how Pro258 interacts with Trp251 in BlaC. Thus, the purpose of Pro258 within class A β‐lactamases may be to secure the loop between the β‐strands B4 and B5, a role that is taken over by Pro257 in TEM‐1.

NMR spectra showed a linear shift for multiple amide resonances of the variants of P107, suggesting that the enzyme samples two states in the fast exchange regime, with each mutant having a different fraction of the two states and, thus, a different average chemical shift. This effect was observed before by other research groups. Jensen and colleagues a reported linear correlation between the chemical shifts of the wild type and two variants of SH3 domain of JIP1 (Perez et al., 2022). They proved that the variants are in fast‐to‐intermediate exchange between two conformations governed by an aromatic ring flip in the protein core. Schütz, Rennella, and Kay showed a peak position of a residue in p97 titrating as a function of mutation in a near‐linear fashion, indicating a rapid exchange between two conformers (Schütz et al., 2017). A similar trend was observed for TEM β‐lactamase by Zimmerman et al., for which resonances were found to display a linear shift pattern from the wild‐type protein and the least stable variants to the most stable variants (Zimmerman et al., 2017). The authors propose that chemical shifts similar to those in the wild‐type enzyme represent nuclei in a more loosely packed interface and those closer to the stabilized variants represent nuclei in a more tightly packed interface. Although the packing of the structure and strength of interaction do not directly explain the shift of the amide resonances in a linear manner, it is possible that the effect observed by Zimmerman and colleagues also indicates the interconversion between different states. In this case, however, the wild‐type enzyme is the one that experiences chemical exchange. The linear shift pattern for various Pro107 mutants is further complicated by line broadening and/or the presence of double peaks for some variants, indicating an exchange between more than two states. We propose a model in which substitutions in Pro107 lead to more flexibility of the region around the mutation site, causing exchange between an open and a closed conformation (fast exchange process, causing the linear shift pattern). Molecules in the open conformation can subsequently interact and dimerize (slow exchange process, causing double peaks), as the two variants BlaC P107A and BlaC P107G with the largest number of double peaks were also the ones with the least monomeric form as detected with SEC‐MALS (Figure 8b). The crystal structure of BlaC P107T illuminated the structure of the dimer. The disorder in the loop region indicates mobility and the formation of the intermolecular β‐sheet shows how dimer formation is stabilized. The dimer formation resembles domain swapping, as the region containing two α‐helices in one protein molecules gets rearranged and the space gets occupied by α‐helices of a second protein molecule. In this case, however, the part that displaces is not the same as the one that gets displaced, as seen in regular domain swapping (Brangulis et al., 2024; Cahyona et al., 2020; Mascarenhas & Gosavi, 2017; Sakai et al., 2023). In a recent study, another β‐lactamase, CTX‐M‐9, was shown to form oligomers as the result of the introduction of two Cys residues, and domain swapping was proposed as a mechanism (Villanueva et al., 2024). It illustrates that small surface changes can enhance homomeric interactions. Our results point toward a role of the conserved prolines in stabilization of the monomer and prevention of aggregation. Such a function of proline residues has been demonstrated for other proteins. In the Fibronectin type III superfamily, it was found that mutating certain conserved prolines did not necessarily result in less activity or any structural changes, but in a shifted equilibrium between monomer and dimer forms (Steward et al., 2002). Comparable results were found in AtbZIP34 and AtbZIP61, which are transcription factors of the bZIP family in *Arabidopsis thaliana* (Shen et al., 2007). These two naturally occurring variants were reported to be unable to form homodimers, contrary to other members from the family. It was found that these two proteins had a single substitution of one of the residues to proline that slightly disrupted the α‐helix structure, thereby disrupting the formation of homodimers. Furthermore, it was shown that substituting certain residues in protein dimers with prolines is a good way to prevent dimerization, due to the inability of the proline nitrogen atom to form H‐bonds (Joseph et al., 2013). Several studies discuss the roles of prolines in the formation of amyloid fibrils (Hasanbašić et al., 2019; Rauscher et al., 2006; Rosenberg et al., 2022). On the other hand, the mutation of Pro28 in insulin to Asp or double mutation Pro28Lys‐Lys29Pro were reported to favor the monomeric state over the naturally occurring hexamers (Brange et al., 1988; Brems et al., 1992).

β‐lactamases are mostly monomeric, however, several OXA enzymes from class D β‐lactamases were found to form dimers (e.g., OXA‐10 (Golemi et al., 2001), OXA‐163 (Lund et al., 2017)). OXA‐14 was shown to form highly active dimers at a high concentration of enzyme, while at a low concentration, the protein was mostly monomeric. It was also demonstrated that metal ions can influence the formation of the dimer for this enzyme. In OXA enzymes dimers are formed by the interaction of β‐sheet domains, different from the interactions in BlaC P107T, and whereas dimer formation is potentially beneficial for the activity of OXA enzyme, this is not the case for BlaC. The plate assays show that cells producing the Pro107 variants have lower β‐lactamase activity than cells producing wild‐type BlaC. The purified enzymes demonstrate a level of activity similar to wild type. Translocation of BlaC is thought to occur in its folded form because the protein has a TAT signal sequence, so it is likely that formation of the dimers or aggregates hinders its targeting to the periplasm.

## 4 CONCLUSIONS

We propose that one of the roles of conserved prolines in BlaC is to prevent protein association and maintain the monomeric state. The data demonstrated that mutations in conserved prolines lead to the preservation of the enzymatic traits, but the long‐term stability of the protein is significantly compromised. BlaC variants were shown to be more prone to aggregation than wild‐type enzyme.

## 5 MATERIALS AND METHODS

### 5.1 Constructs and cells

Site‐directed mutagenesis was performed using whole plasmid site‐directed mutagenesis. Plasmid pUK21 carrying the *blaC* gene, encoding a signal peptide for transmembrane transport, or the plasmid pET28a + carrying the *blaC* gene with the code for a TEV cleavable N‐terminal His(6)‐tag was used as template. *E. coli* KA797 cells transformed with the mutated plasmids were plated on LB agar plates containing 50 μg mL^−1^ kanamycin. The presence of the mutations was confirmed by sequencing, performed by Baseclear BV. For recombinant production of mutant proteins, *E. coli* strain BL21pLysS (DE3) was used in combination with the pET28‐based plasmids. For in cells studies with antibiotics, the KA797 strain was used with pUK21‐based plasmids for constitutive expression.

### 5.2 Protein production and purification

The protein production and purification were done according to previously described protocol (Elings et al., 2017). After the first nickel column samples meant for stability studies or SEC‐MALS experiments were buffer exchanged to the target buffer and the following experiments were performed with the His‐tagged protein to minimize the time of the sample preparation and the effect of multiple buffer exchanges. Wild‐type enzyme was tested both with and without His‐tag and no difference was found for the outcome of the experiments.

For other in vitro experiments the His‐tag was removed by overnight incubation with 0.2 mg mL^−1^ His‐tagged Tobacco Etch Virus (TEV) protease at 4°C in 25 mM Tris‐HCl buffer, pH 8.0 with 100 mM NaCl, 1 mM EDTA and 5 mM DTT, followed by an additional HisTrap Nickel column purification to separate BlaC without His‐tag from uncleaved BlaC and TEV protease. SDS‐PAGE analysis showed single bands of similar sizes for all variant enzymes. The protein concentration was determined by absorption at 280 nm, using the theoretical extinction coefficient 29,910 M^−1^ cm^−1^ (Artimo et al., 2012).

To assess the solubility of the produced enzyme, overnight bacterial cultures induced with 1 mM IPTG were diluted to the same optical density at 600 nm and treated with B‐PER (ThermoFisher Scientific) for 30 min at room temperature. The whole cell lysate sample was taken and part was centrifugated to separate soluble and insoluble fractions. Whole lysate and soluble fraction samples were analyzed using SDS‐PAGE. Bands were visualized with trichloroethanol (Ladner‐Keay et al., 2018).

### 5.3 In cell activity studies

The growth of the *E. coli* cells carrying pUK‐based plasmids with wild‐type or mutant BlaC genes was tested on LB‐agar plates or in liquid cultures with various concentrations of antibiotics. All plates and media contained 50 μg mL^−1^ kanamycin and 1 mM IPTG. Cells were applied on the plates as 10 μL drops with OD_600_ values of 0.3, 0.03, 0.003, or 0.0003. Liquid cultures were grown without β‐lactam antibiotics in duplicate until OD_600_ reached 0.3, at which point they were diluted 200 times with the test media and the recording of OD_600_ was started using BioScreen. Growth was followed in 200 μL culture volumes for 18 h, with the OD_600_ measured every 30 min.

### 5.4 Circular dichroism

CD profiles were recorded using a Jasco J‐815 spectropolarimeter with a Peltier temperature controller (Jasco, MD). Measurements were performed in triplicate at 25°C with 10 μM protein in 100 mM sodium phosphate buffer (pH 6.4). Spectra were acquired in 1 mm quartz cuvette at a scan rate of 50 nm/min.

### 5.5 Thermal stability

Thermal stability of the proteins was analyzed by thermal shift assay with SYPRO Orange dye (Invitrogen) or by tryptophane fluorescence with Tycho NT.6 (Nanotemper). The measurements with SYPRO Orange dye were performed in triplicate in two independent experiments using the CFX 96 Touch Real‐Time PCR Detection System from Bio‐Rad with 2× dye and 10 μM protein in 100 mM sodium phosphate buffer (pH 6.4) with the temperature range 20–80°C. The measurements with Tycho NT.6 were performed in triplicates with 10–20 μM protein in 100 mM sodium phosphate buffer (pH 6.4).

### 5.6 Kinetics

Determination of the Michaelis–Menten kinetic constants was done as described previously (van Alen et al., 2023). All reactions were performed in triplicate in sodium phosphate buffer pH 6.4 at 25°C.

### 5.7 Refolding experiments

For refolding experiments, initial unfolding of BlaC proteins was done using 45 μM of enzyme with 4.5 M guanidinium chloride in 100 mM phosphate buffer (pH 6.4). The mixes were incubated on ice for 20 min to ensure complete unfolding. Refolding at 25°C was initiated by dilution of samples with the same buffer containing 4.5, 2.25, 1.8, 1.125, 0.9, or 0.45 M guanidinium chloride with 100 μM nitrocefin. Untreated BlaC in 100 mM phosphate buffer was used as a positive control. The hydrolysis of nitrocefin was measured at 486 nm on a TECAN Infinite® M1000PRO plate reader. In each measurement, the final BlaC concentration was 0.9 μM. Experiments were carried out in triplicate.

### 5.8 Protein stability measurements

Protein stability over time was assessed as the OD_600_ signal using 10 μM of enzyme solution in either PBS buffer (pH 7.4) or 100 mM sodium phosphate buffer (pH 6.4, 7.5, or 8.5). The process was followed in duplicate at 37°C for 4 h or at 25°C for 22 h with TECAN Infinite® M1000PRO plate reader with measurements performed every minute or every 15 min, respectively.

### 5.9 SEC MALS

The composition of the samples was controlled by SEC with 1260 infinity II LC system (Agilent), using a Superdex 75 increase 10/300 GL column (Cytiva). MALS was measured using a MicroDAWN (Wyatt). Data were analyzed with Astra (Wyatt). The runs were performed with the flow of 0.5 mL/min.

### 5.10 Crystallization

Crystallization conditions for BlaC mutants were screened by sitting‐drop vapor diffusion using the BCS, Morpheus, JCSG+ and PACT premier (Molecular Dimensions) screens at 20°C with 100 nL drops with 1:1 ratio (Newman et al., 2005). The plates were dispensed by the NT8 Drop Setter (Formulatrix). Protein solutions were used with a concentration of 10 mg mL^−1^ in 100 mM sodium phosphate buffer (pH 6.4). Crystal trials were performed for BlaC P226G, BlaC P258A, BlaC P258S, and all BlaC Pro107 variants, however, only BlaC P226G and BlaC P107T yielded crystals suitable for the data collection (conditions can be found in Table 2). The crystals were mounted on cryo‐loops in mother liquor with 25% glycerol as cryoprotectant and vitrified by plunging in liquid nitrogen.

**TABLE 2:** Data collection and refinement statistics.

BlaC variant	P226G	P107T
PDB ID	7A6Z	8R88
Conditions	0.2 M sodium malonate dibasic monohydrate, 20% PEG 3350	0.1 M MB1 (pH 6.5), 0.06 M divalent, 30%w/v 550M_20K
Resolution (Å)	44.72–1.30 (1.32–1.30)	124.59–1.95 (2.00–1.95)
Space group	P 21 21 21	P 1 21 1
Unit cell a, b, c (Å)	53.52, 54.23, 79.05	47.002 70.616124.594
CC_1/2_	99.7 (77.0)	99.5 (84.2)
*R*_pim_ (%)	3.4 (39.3)	6.1 (25.9)
|*I*/σ*I*|	11.0 (1.6)	8.6 (2.4)
Completeness (%)	99.9 (99.9)	99 (96.2)
Multiplicity	6.5	1.9
Unique reflections	57,233	58,924
Refinement		
Atoms protein/ligands/water	2035/67/249	7635/42/472
B‐factors protein/ligands/water	10/23/24	23/44/28
*R*_work_/*R*_free_ (%)	13.1/15.1	16.7/20.7
Bond lengths RMSZ/RMSD (Å)	1.475/ 0.018	0.691/0.012
Bond angles RMSZ/RMSD (°)	1.148/1.873	0.756/1.627
Ramachandran preferred/outliers	249/2	924/8
Clash score	1.84	3.03
MolProbity score	1.08	1.11

*Note*: Numbers in brackets represent the values for the highest resolution shell.

### 5.11 X‐ray data collection, processing, and structure solving

X‐ray diffraction data were obtained from a single crystal at the Swiss Light Source (SLS, Paul Scherrer Institute, Switzerland) for BlaC P226G mutant using an Eiger detector and at the Diamond Light Source (DLS, Oxford, England) for BlaC P107T with a Pilatus detector. The diffraction data extended to a resolution of 1.3 Å for P226G and 1.7 Å for P107T. The resolution was set to 1.95 Å for P107T based on completeness and CC1/2 values. Data were processed and integrated with XDS (Kabsch, 2010) and scaled with AIMLESS (Evans, 2011). The structure was solved by molecular replacement using MOLREP (Winn et al., 2011) from the CCP4 suite (Winn et al., 2011) using PDB entry 2GDN (Wang et al., 2006) as a search model. Building and refinement were performed using Coot and REFMAC (Winn et al., 2011). The model was further optimized using the PDB‐REDO webserver (Joosten et al., 2014). Structure validation showed a RamaZ score (Sobolev et al., 2020) of 0.109 and −0.31, for P226G and P107T respectively; 98%–99% of all residues are within the Ramachandran plot favored regions with two outliers for both structures, namely, Cys69 and Arg220. According to MolProbity (Chen et al., 2010), the structure belongs to the 98th percentile for both structures. Data collection and refinement statistics can be found in Table 2. Crystal structures were deposited in the PDB Database with PDB IDs 7A6Z and 8R88.

### 5.12 NMR spectroscopy experiments

TROSY‐HSQC spectra were recorded on a Bruker AVIII HD 850 MHz spectrometer at 25°C in 100 mM phosphate buffer (pH 6.4) with 6% D_2_O. Data were processed in Topspin 3.2 (Bruker). Spectra were analyzed with CCPNmr Analysis software (versions 2 and 3) (Skinner et al., 2016). Peaks of the mutant spectra were assigned by comparison to peaks in the wild type BlaC spectrum and average chemical shift perturbations (CSP), *Δδ*, of the ^1^H (*Δω*_1_) and ^15^N (*Δω*_2_) resonances of backbone amides were calculated using Equation (1). Peaks that could not be assigned with certainty were assigned based on the smallest possible CSP

(1) $$∆\delta =\sqrt{\frac{1}{2}\left({∆\omega }_{1}^{2}+{\left(\frac{∆\omega_{2}}{5}\right)}^{2}\right)}$$

## AUTHOR CONTRIBUTIONS

**M. Ubbink:** Conceptualization; funding acquisition; writing – review and editing; supervision; project administration. **A. Chikunova:** Conceptualization; investigation; writing – original draft; validation; visualization; methodology. **M. P. Manley:** Investigation. **C. N. Heijjer:** Investigation. **C. S. Drenth:** Investigation. **A. J. Cramer‐Blok:** Investigation. **M. Ud Din Ahmad:** Investigation. **A. Perrakis:** Validation.

## CONFLICT OF INTEREST STATEMENT

The authors declare that they have no conflicts of interest with the contents of this article.

## Supporting information


**Figure S1:** Growth in cultures and on plates of *E. coli* producing various BlaC variants.
**Figure S2:** Results of CD spectroscopy and thermostability assays.
**Figure S3:** Additional results of aggregation assay and SEC MALS.
**Table S1:** Melting temperatures of BlaC variants.
**Table S2:** Molecular weights of BlaC variants detected by SEC MALS.

## References

[pro4972-bib-0001] Adzhubei AA , Sternberg MJE , Makarov AA . Polyproline‐II helix in proteins: structure and function. J Mol Biol. 2013;425:2100–2132. 23507311 10.1016/j.jmb.2013.03.018

[pro4972-bib-0002] Ambler RP , Coulson AFW , Frère JM , Ghuysen JM , Joris B , Forsman M , et al. A standard numbering scheme for the class A β‐lactamases. Biochem J. 1991;276:269–270. 2039479 10.1042/bj2760269 PMC1151176

[pro4972-bib-0003] Armon A , Graur D , Ben‐Tal N . ConSurf: an algorithmic tool for the identification of functional regions in proteins by surface mapping of phylogenetic information. J Mol Biol. 2001;307:447–463. 11243830 10.1006/jmbi.2000.4474

[pro4972-bib-0004] Artimo P , Jonnalagedda M , Arnold K , Baratin D , Csardi G , de Castro E , et al. ExPASy: SIB bioinformatics resource portal. Nucleic Acids Res. 2012;40:597–603. 10.1093/nar/gks400 PMC3394269 22661580

[pro4972-bib-0005] Ashkenazy H , Erez E , Martz E , Pupko T , Ben‐Tal N . ConSurf 2010: calculating evolutionary conservation in sequence and structure of proteins and nucleic acids. Nucleic Acids Res. 2010;38:529–533. 10.1093/nar/gkq399 PMC2896094 20478830

[pro4972-bib-0006] Avci FG , Altinisik FE , Vardar Ulu D , Ozkirimli Olmez E , Sariyar Akbulut B . An evolutionarily conserved allosteric site modulates beta‐lactamase activity. J Enzyme Inhib Med Chem. 2016;31:33–40. 27353461 10.1080/14756366.2016.1201813

[pro4972-bib-0007] Bauer F , Sticht H . A proline to glycine mutation in the Lck SH3‐domain affects conformational sampling and increases ligand binding affinity. FEBS Lett. 2007;581:1555–1560. 17382937 10.1016/j.febslet.2007.03.012

[pro4972-bib-0008] Berezin C , Glaser F , Rosenberg J , Paz I , Pupko T , Fariselli P , et al. ConSeq: the identification of functionally and structurally important residues in protein sequences. Bioinformatics. 2004;20:1322–1324. 14871869 10.1093/bioinformatics/bth070

[pro4972-bib-0009] Bochicchio B , Tamburro AM . Polyproline II structure in proteins: identification by chiroptical spectroscopies, stability, and functions. Chirality. 2002;14:782–792. 12395395 10.1002/chir.10153

[pro4972-bib-0010] Brange J , Ribel U , Hansen JF , Dodson G , Hansen MT , Havelund S , et al. Monomeric insulins obtained by protein engineering and their medical implications. Nature. 1988;333:679–682. 3287182 10.1038/333679a0

[pro4972-bib-0011] Brangulis K , Akopjana I , Bogans J , Kazaks A , Tars K . Structural studies of chromosomally encoded outer surface lipoprotein BB0158 from *Borrelia burgdorferi* sensu stricto. Ticks Tick Born Dis. 2024;15:102287. 10.1016/j.ttbdis.2023.102287 38016210

[pro4972-bib-0012] Brems DN , Alter LA , Beckage MJ , Chance RE , DiMarchi RD , Green LK , et al. Altering the association properties of insulin by amino acid replacement. Protein Eng. 1992;5:527–533. 1438163 10.1093/protein/5.6.527

[pro4972-bib-0013] Cahyona RN , Yamanaka M , Nagao S , Shibata N , Higuchi Y , Hirota S . 3D domain swapping of azurin from Alcaligenes xylosoxidans. Metallomics. 2020;12:337–345. 31956880 10.1039/c9mt00255c

[pro4972-bib-0014] Chen VB , Arendall WB , Headd JJ , Keedy DA , Immormino RM , Kapral GJ , et al. MolProbity: all‐atom structure validation for macromolecular crystallography. Acta Crystallogr D Biol Crystallogr. 2010;66:12–21. 20057044 10.1107/S0907444909042073 PMC2803126

[pro4972-bib-0015] Chikunova A , Ubbink M . The roles of highly conserved, non‐catalytic residues in class A β‐lactamases. Protein Sci. 2022;31:e4328. 35634774 10.1002/pro.4328 PMC9112487

[pro4972-bib-0016] Cook KH , Schmid FX , Baldwin RL . Role of proline isomerization in folding of ribonuclease A at low temperatures. Proc Natl Acad Sci USA. 1979;76:6157–6161. 293712 10.1073/pnas.76.12.6157 PMC411822

[pro4972-bib-0017] del Sol MA , Pazos F , Valencia A . Automatic methods for predicting functionally important residues. J Mol Biol. 2003;326:1289–1302. 12589769 10.1016/s0022-2836(02)01451-1

[pro4972-bib-0018] Elings W , Tassoni R , van der Schoot SA , Luu W , Kynast JP , Dai L , et al. Phosphate promotes the recovery of *Mycobacterium tuberculosis* β‐lactamase from clavulanic acid inhibition. Biochemistry. 2017;56:6257–6267. 29087696 10.1021/acs.biochem.7b00556 PMC5707625

[pro4972-bib-0019] Evans PR . An introduction to data reduction: space‐group determination, scaling and intensity statistics. Acta Crystallogr D Biol Crystallogr. 2011;67:282–292. 21460446 10.1107/S090744491003982X PMC3069743

[pro4972-bib-0020] Feiler C , Fisher AC , Boock JT , Marrichi MJ , Wright L , Schmidpeter PAM , et al. Directed evolution of *Mycobacterium tuberculosis* β‐lactamase reveals gatekeeper residue that regulates antibiotic resistance and catalytic efficiency. PLoS One. 2013;8:e73123. 24023821 10.1371/journal.pone.0073123 PMC3762836

[pro4972-bib-0021] Fryszczyn BG , Adamski CJ , Brown NG , Rice K , Huang W , Palzkill T . Role of β‐lactamase residues in a common interface for binding the structurally unrelated inhibitory proteins BLIP and BLIP‐II. Protein Sci. 2014;23:1235–1246. 24947275 10.1002/pro.2505 PMC4243995

[pro4972-bib-0022] Golemi D , Maveyraud L , Vakulenko S , Samama JP , Mobashery S . Critical involvement of a carbamylated lysine in catalytic function of class D beta‐lactamases. Proc Natl Acad Sci USA. 2001;98:14280–14285. 11724923 10.1073/pnas.241442898 PMC64673

[pro4972-bib-0023] Gupta S , Purohit P , Auerbach A . Function of interfacial prolines at the transmitter‐binding sites of the neuromuscular acetylcholine receptor. J Biol Chem. 2013;288:12667–12679. 23519471 10.1074/jbc.M112.443911 PMC3642313

[pro4972-bib-0024] Hasanbašić S , Taler‐Verčič A , Puizdar V , Stoka V , Tušek Žnidarič M , Vilfan A , et al. Prolines affect the nucleation phase of amyloid fibrillation reaction; mutational analysis of human stefin B. ACS Chem Nerosci. 2019;10:2730–2740. 10.1021/acschemneuro.8b00621 PMC6727212 30924329

[pro4972-bib-0025] Hiniker A , Vertommen D , Bardwell JCA , Collet J‐F . Evidence for conformational changes within DsbD: possible role for membrane‐embedded proline residues. J Bacteriol. 2006;188:7317–7320. 17015672 10.1128/JB.00383-06 PMC1636233

[pro4972-bib-0026] Hujer AM , Bethel CR , Bonomo RA . Antibody mapping of the linear epitopes of CMY‐2 and SHV‐1 β‐lactamases. Antimicrob Agents Chemother. 2004;48:3980–3988. 15388462 10.1128/AAC.48.10.3980-3988.2004 PMC521864

[pro4972-bib-0027] Joosten RP , Long F , Murshudov GN , Perrakis A . The PDB‐REDO server for macromolecular structure model optimization. IUCrJ. 2014;1:213–220. 10.1107/S2052252514009324 PMC4107921 25075342

[pro4972-bib-0028] Joseph PRB , Poluri KM , Gangavarapu P , Rajagopalan L , Raghuwanshi S , Richardson RM , et al. Proline substitution of dimer interface β‐strand residues as a strategy for the design of functional monomeric proteins. Biophys J. 2013;105:1491–1501. 24048001 10.1016/j.bpj.2013.08.008 PMC3785864

[pro4972-bib-0029] Joshi AD , Pajor AM . Role of conserved prolines in the structure and function of the Na+/dicarboxylate cotransporter 1, NaDC1. Biochemistry. 2006;45:4231–4239. 16566597 10.1021/bi052064y PMC2547120

[pro4972-bib-0030] Kabsch W . XDS. Acta Crystallogr Sect D: Biol Crystallogr. 2010;66:125–132. 20124692 10.1107/S0907444909047337 PMC2815665

[pro4972-bib-0031] Kemper B . Structural basis for the role in protein folding of conserved proline‐rich regions in cytochromes P450. Toxicol Appl Pharmacol. 2004;199:305–315. 15364546 10.1016/j.taap.2003.11.030

[pro4972-bib-0032] Kini RM , Evans HJ . A hypothetical structural role for proline residues in the flanking segments of protein–protein interaction sites. Biochem Biophys Res Commun. 1995;212:1115–1124. 7626100 10.1006/bbrc.1995.2084

[pro4972-bib-0033] Krieger F , Möglich A , Kiefhaber T . Effect of proline and glycine residues on dynamics and barriers of loop formation in polypeptide chains. J Am Chem Soc. 2005;127:3346–3352. 15755151 10.1021/ja042798i

[pro4972-bib-0034] Ladner‐Keay CL , Turner RJ , Edwards RA . Fluorescent protein visualization immediately after gel electrophoresis using an in‐gel Trichloroethanol photoreaction with tryptophan. Methods Mol Biol. 2018;1853:179–190. 30097944 10.1007/978-1-4939-8745-0_22

[pro4972-bib-0035] Levitt M . Effect of proline residues on protein folding. J Mol Biol. 1981;145:251–263. 7265199 10.1016/0022-2836(81)90342-9

[pro4972-bib-0036] Lund BA , Thomassen AM , Carlsen TJO , Leiros HKS . Structure, activity and thermostability investigations of OXA‐163, OXA‐181 and OXA‐245 using biochemical analysis, crystal structures and differential scanning calorimetry analysis. Acta Crystallogr F Struct Biol Commun. 2017;73:579–587. 28994407 10.1107/S2053230X17013838 PMC5633926

[pro4972-bib-0037] MacArthur MW , Thornton JM . Influence of proline residues on protein conformation. J Mol Biol. 1991;218:397–412. 2010917 10.1016/0022-2836(91)90721-h

[pro4972-bib-0038] Mascarenhas NM , Gosavi S . Understanding protein domain‐swapping using structure‐based models of protein folding. Prog Biophys Mol Biol. 2017;128:113–120. 27867057 10.1016/j.pbiomolbio.2016.09.013 PMC7127520

[pro4972-bib-0039] McDonald CB , Seldeen KL , Deegan BJ , Farooq A . SH3 domains of Grb2 adaptor bind to PXpsiPXR motifs within the Sos1 nucleotide exchange factor in a discriminate manner. Biochemistry. 2009;48:4074–4085. 19323566 10.1021/bi802291y PMC2710136

[pro4972-bib-0040] Meneksedag D , Dogan A , Kanlikilicer P , Ozkirimli E . Communication between the active site and the allosteric site in class A beta‐lactamases. Comput Biol Chem. 2013;43:1–10. 23314151 10.1016/j.compbiolchem.2012.12.002

[pro4972-bib-0041] Morgan AA , Rubenstein E . Proline: the distribution, frequency, positioning, and common functional roles of proline and polyproline sequences in the human proteome. PLoS One. 2013;8:e53785. 23372670 10.1371/journal.pone.0053785 PMC3556072

[pro4972-bib-0042] Newman J , Egan D , Walter TS , Meged R , Berry I , Ben JM , et al. Towards rationalization of crystallization screening for small‐ to medium‐sized academic laboratories: the PACT/JCSG+ strategy. Acta Crystallogr D Biol Crystallogr. 2005;61:1426–1431. 16204897 10.1107/S0907444905024984

[pro4972-bib-0043] Palmieri F , Pierri CL . Structure and function of mitochondrial carriers—role of the transmembrane helix P and G residues in the gating and transport mechanism. FEBS Lett. 2010;584:1931–1939. 19861126 10.1016/j.febslet.2009.10.063

[pro4972-bib-0044] Perez LM , Fs I , Bessa LM , Maurin D , Kragelj J , Blackledge M , et al. Visualizing protein breathing motions associated with aromatic ring flipping. Nature. 2022;602:695–700. 35173330 10.1038/s41586-022-04417-6 PMC8866124

[pro4972-bib-0045] Peterson FC , Volkman BF . Diversity of polyproline recognition by EVH1 domains. Front Biosci (Landmark Ed). 2009;14:833–846. 19273103 10.2741/3281 PMC3882067

[pro4972-bib-0046] Rauscher S , Baud S , Miao M , Keeley FW , Pomès R . Proline and glycine control protein self‐organization into elastomeric or amyloid fibrils. Structure. 2006;14:1667–1676. 17098192 10.1016/j.str.2006.09.008

[pro4972-bib-0047] Rosenberg GM , Murray KA , Salwinski L , Hughes MP , Abskharon R , Eisenberg DS . Bioinformatic identification of previously unrecognized amyloidogenic proteins. J Biol Chem. 2022;298:101920. 35405097 10.1016/j.jbc.2022.101920 PMC9108986

[pro4972-bib-0048] Rudgers GW , Palzkill T . Identification of residues in β‐lactamase critical for binding β‐ lactamase inhibitory protein. J Biol Chem. 1999;274:6963–6971. 10066750 10.1074/jbc.274.11.6963

[pro4972-bib-0049] Sakai T , Mashima T , Kobayashi N , Ogata H , Duan L , Fujiki R , et al. Structural and thermodynamic insights into antibody light chain tetramer formation through 3D domain swapping. Nat Commun. 2023;14:7807. 38065949 10.1038/s41467-023-43443-4 PMC10709643

[pro4972-bib-0050] Schmid FX , Baldwin RL . Acid catalysis of the formation of the slow‐folding species of RNase A: evidence that the reaction is proline isomerization. Proc Natl Acad Sci USA. 1978;75:4764–4768. 283390 10.1073/pnas.75.10.4764 PMC336200

[pro4972-bib-0051] Schuster‐Böckler B , Schultz J , Rahmann S . HMM logos for visualization of protein families. BMC Bioinformatics. 2004;5:7. 14736340 10.1186/1471-2105-5-7 PMC341448

[pro4972-bib-0052] Schütz AK , Rennella E , Kay LE . Exploiting conformational plasticity in the AAA+ protein VCP/p97 to modify function. Proc Natl Acad Sci USA. 2017;114:E6822–E6829. 28760999 10.1073/pnas.1707974114 PMC5565461

[pro4972-bib-0053] Shen H , Cao K , Wang X . A conserved proline residue in the leucine zipper region of AtbZIP34 and AtbZIP61 in Arabidopsis thaliana interferes with the formation of homodimer. Biochem Biophys Res Commun. 2007;362:425–430. 17719007 10.1016/j.bbrc.2007.08.026

[pro4972-bib-0054] Skinner SP , Fogh RH , Boucher W , Ragan TJ , Mureddu LG , Vuister GW . CcpNmr AnalysisAssign: a flexible platform for integrated NMR analysis. J Biomol NMR. 2016;66:111–124. 27663422 10.1007/s10858-016-0060-y PMC5095159

[pro4972-bib-0055] Sobolev OV , Afonine PV , Moriarty NW , Hekkelman ML , Joosten RP , Perrakis A , et al. A global Ramachandran score identifies protein structures with unlikely stereochemistry. Structure. 2020;28:1249–1258.e2. 32857966 10.1016/j.str.2020.08.005 PMC7642142

[pro4972-bib-0056] Stec B , Holtz KM , Wojciechowski CL , Kantrowitz ER . Structure of the wild‐type TEM‐1 beta‐lactamase at 1.55 A and the mutant enzyme Ser70Ala at 2.1 A suggest the mode of noncovalent catalysis for the mutant enzyme. Acta Crystallogr D Biol Crystallogr. 2005;61:1072–1079. 16041072 10.1107/S0907444905014356

[pro4972-bib-0057] Steward A , Adhya S , Clarke J . Sequence conservation in Ig‐like domains: the role of highly conserved proline residues in the fibronectin type III superfamily. J Mol Biol. 2002;318:935–940. 12054791 10.1016/S0022-2836(02)00184-5

[pro4972-bib-0058] Valdar WSJ . Scoring residue conservation. Proteins. 2002;48:227–241. 12112692 10.1002/prot.10146

[pro4972-bib-0059] van Alen I , Chikunova A , van Zanten DB , de Block AA , Timmer M , Brunle S , et al. Asp179 in the class A β ‐lactamase from *Mycobacterium tuberculosis* is a conserved yet not essential residue due to epistasis. FEBS J. 2023;290:4933–4949. 37335937 10.1111/febs.16892

[pro4972-bib-0060] van Arnam EB , Lester HA , Dougherty DA . Dissecting the functions of conserved prolines within transmembrane helices of the D2 dopamine receptor. ACS Chem Biol. 2011;6:1063–1068. 21776983 10.1021/cb200153g PMC3199346

[pro4972-bib-0061] Villanueva M , Vostal LE , Cohen DN , Biesbrock D , Kuwaye EP , Driver SG , et al. Differential effects of disulfide bond formation in TEM‐1 versus CTX‐M‐9 β‐lactamase. Protein Sci. 2024;33:e4816. 37897253 10.1002/pro.4816 PMC10731493

[pro4972-bib-0062] Wang F , Cassidy C , Sacchettini JC . Crystal structure and activity studies of the *Mycobacterium tuberculosis* β‐lactamase reveal its critical role in resistance to β‐lactam antibiotics. Antimicrob Agents Chemother. 2006;50:2762–2771. 16870770 10.1128/AAC.00320-06 PMC1538687

[pro4972-bib-0063] Williamson MP . The structure and function of proline‐rich regions in proteins. Biochem J. 1994;297:249–260. 8297327 10.1042/bj2970249 PMC1137821

[pro4972-bib-0064] Winn MD , Ballard CC , Cowtan KD , Dodson EJ , Emsley P , Evans PR , et al. Overview of the CCP4 suite and current developments. Acta Crystallogr D Biol Crystallogr. 2011;67:235–242. 21460441 10.1107/S0907444910045749 PMC3069738

[pro4972-bib-0065] Zimmerman MI , Hart KM , Sibbald CA , Frederick TE , Jimah JR , Knoverek CR , et al. Prediction of new stabilizing mutations based on mechanistic insights from Markov state models. ACS Cent Sci. 2017;3:1311–1321. 29296672 10.1021/acscentsci.7b00465 PMC5746865

