# Supplementary material for: Conserved proline residues prevent dimerization and aggregation in the β‐lactamase BlaC
Source: Protein Sci. 2024 Mar 27;33(4):e4972. doi: 10.1002/pro.4972 (PMC10966351; doi:10.1002/pro.4972)
Supplement: Supplementary file 1 — Figure S1: Growth in cultures and on plates of E. coli producing various BlaC variants. Figure S2: Results of CD spectroscopy and thermostability assays. Figure S3: Additional results of aggregation assay and SEC MALS. Table S1: Melting temperatures of BlaC variants. Table S2: Molecular weights of BlaC variants detected by SEC MALS. [file PRO-33-e4972-s001.docx]

Supporting information to

**Conserved proline residues prevent dimerization and aggregation in the β-lactamase BlaC**

A. Chikunova, M. P. Manley, C. N. Heijer, C. S. Drenth, A. J. Cramer-Blok, M. Ud Din Ahmad, A. Perrakis, M. Ubbink


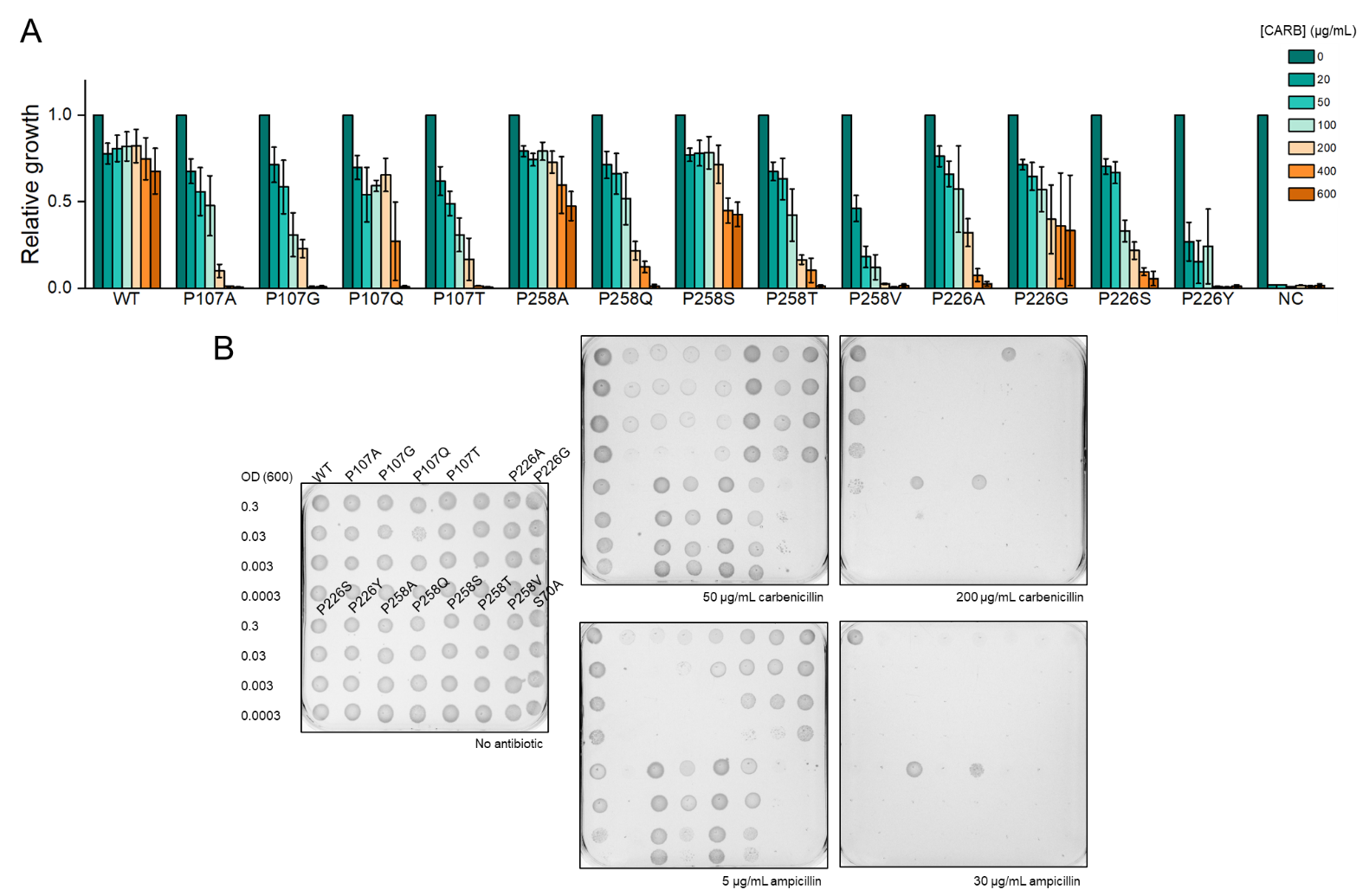


**Figure S1.** Growth in cultures and on plates. (A) Growth of E. coli cells in liquid cultures in presence of different concentrations of carbenicillin relative to growth without antibiotic, determined as OD of liquid cultures after 18 hours incubation at 37 ˚C. Error bars represent standard deviation of two biological replicates; (B) Plates showing growth at 37 ˚C of E. coli cells expressing wild type or mutant blaC genes with no antibiotics or in presence of ampicillin or carbenicillin. The unlabeled culture is a P226A culture contaminated with wild type BlaC culture and these results must be disregarded.


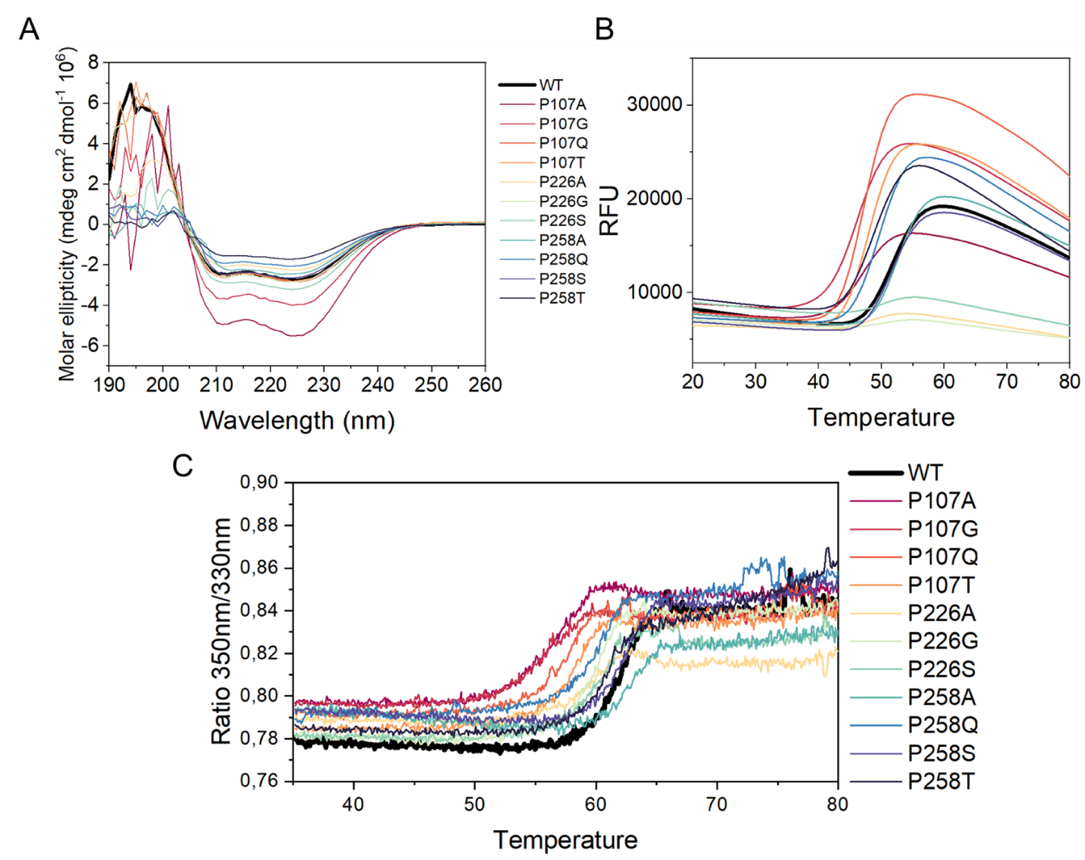


**Figure S2.** CD analysis and thermostability. (A) Circular dichroism spectra of BlaC variants; (B, C) Melting profiles of BlaC variants in the assay with a hydrophobic dye (B) or tryptophane fluorescence (C).


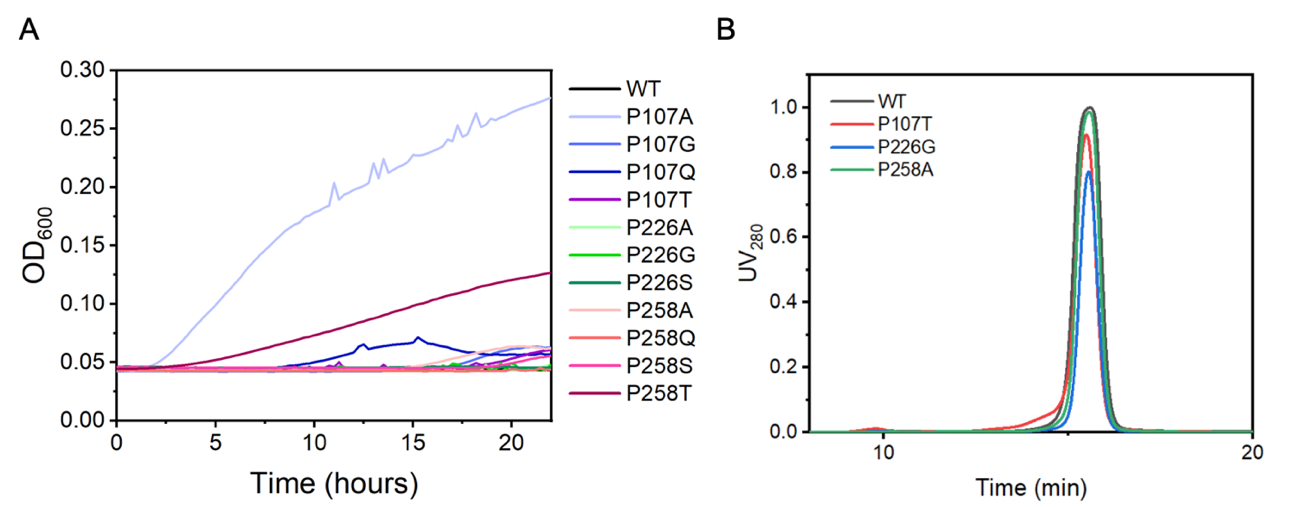


**Figure S3.** (A) Formation of aggregates followed by absorbance at 600 nm at 25 ˚C in PBS buffer; (B) SEC profiles of wild type BlaC and BlaC variants in 100 mM sodium phosphate buffer, pH 6.4, with the column equilibrated with the same buffer.

**Table S1.** Melting temperatures determined with two different methods. Errors represent the standard deviation of a triplicate measurement.

|  | Thermostability  (Trp fluorescence) | Thermostability  (Hydrophobic dye) |
| --- | --- | --- |
|  | Tm (˚C) | Tm (˚C) |
| WT | 62.0 ± 0.1 | 51.92 ± 0.03 |
| P107A | 55.9 ± 0.1 | 46.9 ± 0.1 |
| P107G | 55.7 ± 0.1 | 45.8 ± 0.1 |
| P107Q | 57.4 ± 0.1 | 47.4 ± 0.3 |
| P107T | 57.92 ± 0.02 | 48.0 ± 0.1 |
| P226A | 59.1 ± 0.1 | 47.9 ± 0.2 |
| P226G | 59.8 ± 0.1 | 50.3 ± 0.1 |
| P226S | 59.9 ± 0.1 | 49.9 ± 0.3 |
| P258A | 63.6 ± 0.2 | 52.7 ± 0.1 |
| P258Q | 60.6 ± 0.2 | 49.4 ± 0.1 |
| P258S | 62.7 ± 0.1 | 51.77 ± 0.01 |
| P258T | 60.8 ± 0.1 | 48.5 ± 0.1 |

**Table S2**. Example of molecular weights in kDa determined via SEC-MALS with refractive index for different peaks of BlaC variants for one of the measurements. Samples were stored in 100 mM sodium phosphate buffer (pH 6.4), and the column was equilibrated with PBS buffer (pH 7.5). Errors represent the certainty of the calculation as given by a software.

| BlaC variant | Peak#1 | Peak#2 | Peak#3 | Peak#4 | Peak#5 |
| --- | --- | --- | --- | --- | --- |
| WT | 28 ± 0% | 49 ± 6% |  |  |  |
| P107A | 42 ± 5% | 49 ± 2% | 65 ± 2% | 86 ± 3% | 27780 ± 4% |
| P107G | 48 ± 11% | 54 ± 4% | 87 ± 2% | 190 ± 2% | 10770 ± 7% |
| P107Q | 49 ± 10% | 59 ± 5% | 88 ± 4% | 205 ± 17% | 29330 ± 7% |
| P107T | 50 ± 2% | 65 ± 2% | 98 ± 2% | 56810 ± 4% |  |
| P226A | 35 ± 7% | 70 ± 11% | 162 ± 8% |  |  |
| P226G | 32 ± 2% | 53 ± 2% | 71 ± 8% | 13420 ± 16% |  |
| P226S | 34 ± 2% | 68 ± 3% | 13540 ± 7% |  |  |
| P258A | 37 ± 9% | 58760 ± 57% |  |  |  |
| P258S | 35 ± 9% | 410 ± 47% |  |  |  |
| P258T | 38 ± 11% | 146 ± 32% | 1407 ± 35% | 67590 ± 6% |  |
